# Characteristics of a loop of evidence that affect detection and estimation of inconsistency: a simulation study

**DOI:** 10.1186/1471-2288-14-106

**Published:** 2014-09-19

**Authors:** Areti Angeliki Veroniki, Dimitris Mavridis, Julian PT Higgins, Georgia Salanti

**Affiliations:** Department of Hygiene and Epidemiology, University of Ioannina School of Medicine, University Campus, Ioannina 45110 Greece; Department of Primary Education, University of Ioannina, Ioannina, Greece; School of Social and Community Medicine, University of Bristol, Bristol, UK; Centre for Reviews and Dissemination, University of York, York, UK

**Keywords:** Mixed treatment comparison, Multiple interventions, Coherence, Consistency, Simulation study, Bias

## Abstract

**Background:**

The assumption of consistency, defined as agreement between direct and indirect sources of evidence, underlies the increasingly popular method of network meta-analysis. This assumption is often evaluated by statistically testing for a difference between direct and indirect estimates within each loop of evidence. However, the test is believed to be underpowered. We aim to evaluate its properties when applied to a loop typically found in published networks.

**Methods:**

In a simulation study we estimate type I error, power and coverage probability of the inconsistency test for dichotomous outcomes using realistic scenarios informed by previous empirical studies. We evaluate test properties in the presence or absence of heterogeneity, using different estimators of heterogeneity and by employing different methods for inference about pairwise summary effects (Knapp-Hartung and inverse variance methods).

**Results:**

As expected, power is positively associated with sample size and frequency of the outcome and negatively associated with the presence of heterogeneity. Type I error converges to the nominal level as the total number of individuals in the loop increases. Coverage is close to the nominal level in most cases. Different estimation methods for heterogeneity do not greatly impact on test performance, but different methods to derive the variances of the direct estimates impact on inconsistency inference. The Knapp-Hartung method is more powerful, especially in the absence of heterogeneity, but exhibits larger type I error. The power for a ‘typical’ loop (comprising of 8 trials and about 2000 participants) to detect a 35% relative change between direct and indirect estimation of the odds ratio was 14% for inverse variance and 21% for Knapp-Hartung methods (with type I error 5% in the former and 11% in the latter).

**Conclusions:**

The study gives insight into the conditions under which the statistical test can detect important inconsistency in a loop of evidence. Although different methods to estimate the uncertainty of the mean effect may improve the test performance, this study suggests that the test has low power for the ‘typical’ loop. Investigators should interpret results very carefully and always consider the comparability of the studies in terms of potential effect modifiers.

**Electronic supplementary material:**

The online version of this article (doi:10.1186/1471-2288-14-106) contains supplementary material, which is available to authorized users.

## Background

The validity of results from network meta-analysis depends on the plausibility of the transitivity assumption; that is the comparability of studies informing the treatment comparisons with respect to the distribution of effect modifiers [1–3]. Lack of transitivity in a network can create statistical disagreement between direct and various sources of indirect evidence, often termed inconsistency [4]. Statistical evaluation of consistency is possible only when there are ‘closed loops of evidence’ in the network. The recent increase in applications of network meta-analysis has emphasised the need for methods to evaluate consistency and has motivated the development of statistical models [5–7] and methods [8–11].

Empirical evidence suggests that the prevalence of statistically significant loop inconsistency ranges from 2% to 17% [12–14]. However, little is known about factors that impact on the detection of inconsistency. As expected, the power to detect inconsistency is positively associated with the number and size of trials, and both power and type I error increase when a fixed-effect model is assumed [15]. It has been argued that the presence and magnitude of heterogeneity (within comparison variability) in a loop of evidence can impact on inferences made about inconsistency and empirical evidence has confirmed these claims by showing that different estimators of the heterogeneity variance are likely to have a considerable impact [14]. Finally, previous studies showed that inconsistency occurs more frequently in loops where one of the comparisons is informed only by one trial [14, 16, 17].

Although there are indications that the presence, magnitude and estimation method of heterogeneity might influence the detection of inconsistency, this association has not been studied extensively. For instance, the impact of two alternative methods to express uncertainty about the pairwise summary effects (inverse variance and Knapp-Hartung method [18, 19]) remains unclear. It has been shown that the Knapp-Hartung method outperforms inverse variance in coverage for the summary effect and that it is insensitive to the estimator of the heterogeneity used [20, 21]. We anticipate that differences in the properties of the two methods will impact on the estimation of inconsistency.

The aim of this paper is to explore factors that affect the detection of inconsistency in a three-treatment network for a dichotomous outcome. The factors that we explore are associated with the amount of data available in the loop (such as number, size and distribution of trials across comparisons, frequency of events), the heterogeneity variance in the pairwise comparisons (presence or absence and estimation method) and the method for inference about pairwise summary effects (inverse variance or Knapp-Hartung). We consider only log-odds ratio (LOR) as the effect size of interest. We conduct a simulation study considering realistic scenarios including only two-arm trials and we estimate type I error, power and coverage probability for the test of consistency. The simulation scenarios are informed by two previous empirical studies; a large collection of 303 loops from published networks of interventions [14] and a study about the empirical distribution of heterogeneity on dichotomous outcomes [22].

## Methods

### The inconsistency test

Consider a simple scenario with three competing treatments A, B and C and that there are trials that compare directly all three possible pairs of treatments. Evaluation of inconsistency in a triangular network requires first the estimation of three direct summary effects for each pairwise comparison. We denote the effect sizes (i.e. LORs) for the three pairs of treatments as  and  with variances  and  respectively. The superscript denotes the source of evidence (‘DIR’ for direct here or ‘IND’ indirect later) and the subscript denotes the treatment comparison. For any given comparison (e.g. BC) we estimate the indirect mean treatment effect, , as a simple contrast of two direct estimates involving the third treatment, and we compare it with the corresponding direct estimate .

The inconsistency factor (IF) for the loop ABC is estimated as


with variance
1

The direction of the estimated IF is irrelevant to the evaluation of inconsistency and only the magnitude of its absolute value is of interest. The subscript in  refers to the loop in which inconsistency is estimated.

Under the null hypothesis of consistency (H_0_ : IF = 0) a z-test is calculated


with a critical region |z| ≥ z_*a*/2_. In the present study we select *a* = 0.05.

### Estimation of variance

Equation (1) suggests that the method used to estimate the variance of the direct treatment effects  and  will play an important role in the performance of the z-test for inconsistency. We consider two methods to estimate the direct variances and examine how they can impact on the estimation of . The first method is the usual inverse-variance method and the second method is an alternative approach proposed by Knapp and Hartung [19].

In a pairwise meta-analysis we either assume that trials estimate a single underlying effect size (fixed-effect model) or that the study-specific underlying effect sizes are different but drawn from the same distribution (random effects model) with heterogeneity τ^2^. Under the latter scenario, it is common to assume that heterogeneity is the same for all comparisons being made, i.e. . We adopt this assumption throughout the paper and we estimate τ^2^ using the DerSimonian and Laird estimator [23].

In the inverse variance approach, the direct variances are simple functions of the sampling variances of the individual trials and the heterogeneity variance τ^2^. Suppose that K_AB_, K_AC_ and K_BC_ trials inform the AB, AC and BC comparisons respectively. If the sampling variances were the same for all trials (σ^2^), the inverse variance estimator of the inconsistency variance would be
2

Consequently,  depends on the heterogeneity and decreases with the number and precision of the included trials.

An alternative approach to estimate each direct variance, and consequently , is the approach proposed by Knapp and Hartung [19]. They derive the variance  as the ratio of a generalised Q statistic divided by the product of the degrees of freedom (K_AB_ - 1) and the sum of the random-effects study weights [24]. It has been shown that the performance of this method is not influenced by the choice of the heterogeneity estimator [19, 21, 25, 26].

In summary, we estimate the variances of the direct pairwise summary effects by employing two different strategies: the inverse variance method using DerSimonian and Laird estimator (IVDL) and the Knapp-Hartung method with the DerSimonian and Laird estimator (KHDL). When a comparison is addressed by a single trial (so that the loop includes 3 trials in total) estimation of heterogeneity is impossible. In these cases we use the fixed-effect model (by setting τ^2^ to be zero) and consequently both IVDL and KHDL methods would yield exactly the same results.

### Simulation study

#### Empirical evidence to inform simulation scenarios

**Figure 1:**
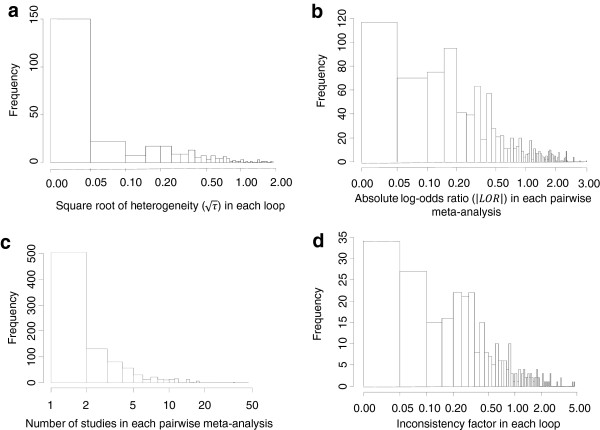
**Histograms of the within-loop heterogeneity, mean treatment effect, number of trials per meta-analysis and inconsistency.** Mean treatment effects are displayed on the absolute of the log-odds ratio scale. Heterogeneity is estimated with the DerSimonian and Laird method. Histograms are plotted for 40 published networks of evidence [14].

To inform the simulation scenarios we use a large collection of complex networks of interventions [14]. Figure 1 summarises some of the attributes of 303 loops from 40 published networks with dichotomous outcomes analysed using the LOR scale. The majority of the pairwise meta-analyses (93%) included fewer than ten trials. The median |LOR| is 0.32 with interquartile range (IQR) (0.13, 0.75). In 91% of the loops the common within-loop heterogeneity using the DerSimonian and Laird estimator is less than 0.5 and it is estimated at zero (when rounded to the second decimal) in 51% of the loops. The median IF is 0.36 with IQR (0.15, 0.80). The median number of trials per loop is 8 IQR (6, 14) and the median loop sample size is 2256 IQR (1026, 18890); the respective median number of trials and sample size per comparison are 2 IQR (1, 4) and 706 IQR (255, 2997). Most networks had a subjective primary outcome (43%), whereas 35% and 22% of the networks had reasonably objective outcomes (e.g. cause-specific mortality, major morbidity event) and all-cause mortality outcomes respectively. The majority of the networks (63%) compared pharmacological interventions versus placebo. In the case of such a comparison type and subjective outcome, Turner et al. suggest that the distribution of the heterogeneity is reasonably approximated by a log-normal τ^2^ ~ LN(-2.13, 1.58^2^), with median τ^2^ = 0.12 and IQR (0.03, 0.34) [22]. Our empirical data seem to match the predictive distribution suggested by Turner et al. [22] (τ^2^ ~ LN(-2.13, 1.58^2^)), though more data are needed since we have only 55 common within-loop heterogeneities estimated in networks with pharmacological interventions versus placebo comparison type and subjective outcome.

#### Simulation scenarios

We use subscripts k_1_, k_2_ and k_3_ to refer to the three comparisons AB, AC and BC respectively, so that k_1_ = 1, …, K_AB_, k_2_ = 1, …, K_AC_ and k_3_ = 1, …, K_BC_, where K_AB_, K_AC_, K_BC_ represent the number of trials included in AB, AC and BC comparisons respectively. We examine both balanced direct comparisons, i.e. all comparisons include the same number of trials K_AB_ = K_AC_ = K_BC_ = K = 1, …, 7, and imbalanced direct comparisons, i.e. each comparison is informed by a different number of trials with K_AB_ = 1, K_AC_ = 4, K_BC_ = 7. Both balanced and imbalanced scenarios were selected, informed by the empirical data. In particular, the imbalanced scenario included a comparison with a single trial, because the majority (196 out of 303) of observed loops had this characteristic. We then set the second comparison to include a large number of trials (7 trials) and for the third comparison we selected the median between the two extremes (4 trials). We restrict our analysis to dichotomous outcome data measured using odds-ratio (OR) due to its mathematical properties [27–29]. Based on the results from the empirical study [14], we assume OR_AB_ = 1/exp(0.32) = 0.73 and OR_AC_ = 1 the relative treatment effects for AB and AC respectively. We compute the OR for the BC comparison as


We select values IF_ABC_ = {0, 0.3, 0.45, 0.6, 1} to cover a range of plausible values for inconsistency as suggested by empirical data (Figure 1d). We consider two different distributions for heterogeneity that pertain to a subjective outcome (the most frequently reported outcome in our data) and all-cause mortality for comparisons between pharmacological interventions and placebo; according to [22] these are τ^2^ ~ LN(-2.13, 1.58^2^) and τ^2^ ~ LN(-4.06, 1.45^2^) (median τ^2^ = 0.02 with (IQR 0.01, 0.04)).

For each combination of OR, IF_ABC_, and τ^2^ we simulate the trial-specific underlying relative treatment effects from a normal distribution as

,  and 

Then, we generate arm-level data for each trial k_1_, k_2_ and k_3_. Without loss of generality we describe how to obtain arm-level data for an AB trial. We assume equal sample sizes across arms, that is . The observed IQR for arm sample size in our empirical data is 51 to 270, and to represent moderate and large studies we generated studies with n ~ U(50, 150) and n ~ U(150, 300). We also considered n ~ U(20, 50) to generate data for very small studies. The number of events per arm, denoted with  and  are drawn from two binomial distributions  and  where  and  are the probabilities of the outcome in each trial arm. To define these probabilities we make assumptions about the average risk (AR) of the outcome in the trial assuming both frequent and rare events. To simulate from frequent event rates we draw from a uniform distribution  and for rare events 

Then the event probabilities in the arms are obtained as the solution to the equations


For frequent events and assuming no heterogeneity, the expected mean variance of LOR ranges from 0.04 to 0.25 depending on sample size. Variances for LOR for rare events range from 0.10 to 0.69.

We then calculate the sample LOR and its variance as


If the simulated number of events in one of the study arms is zero, we add 0.5 to the cells of the 2 × 2 table. We repeat this process for all K_AB_ trials and then we perform a random-effects meta-analysis to obtain the summary effect size . We follow the same process for comparisons AC and BC and then we estimate the inconsistency factor. Table 1 presents a summary of the simulation scenarios considered.

**Table 1:** **Summary of the simulation scenarios**

**Number of studies**	
Balanced direct comparisons	K_AB_ = K_AC_ = K_BC_ = 1, …, 7
Imbalanced direct comparisons	K_AB_ = 1, K_AC_ = 4, K_BC_ = 7 (and K_AB_ = 1, K_AC_ = 4, K_BC_ = 3 for the typical loop)
**Treatment effects**	
Comparison AB	OR_AB_ = 0.73
Comparison AC	OR_AC_ = 1
Comparison BC	OR_BC_ = exp{log(OR_AC_) - log(OR_AB_) + IF_ABC_}
**Inconsistency in the network**	
Inconsistency Factor	IF_ABC_ = {0, 0.3, 0.45, 0.6, 1}
**Heterogeneity in the network**	
Subjective outcome	τ^2^ ~ LN(-2.13, 1.58^2^)
All-cause mortality outcome	τ^2^ ~ LN(-4.06, 1.45^2^)
**Trial arm size**	
Small	n ~ U(20, 50)
Moderate	n ~ U(50, 150)
Large	n ~ U(150, 300) (and n ~ U(120, 160) for the typical loop)
**Frequency of events**	
Average risk for frequent events	
Average risk for rare events	
**Approaches to estimate the variances of the direct pairwise summary effects**	
Inverse variance method	
Knapp-Hartung method	

For each scenario we analyse 1000 simulated triangular networks. Assuming a 5% significance level, we estimate the power of the test when true inconsistency is present (P(|z| ≥ 1.96|IF ≠ 0) and type I error when the null hypothesis is true (P(|z| ≥ 1.96|IF = 0). We compute the coverage probability for the confidence interval (CI) of inconsistency, which is the probability that the estimated interval for IF includes its true value. We carry out the simulations in the freely available software R 2.15.2 [30] using the self-programmed *sims.fun* function, which we have made available online (http://www.mtm.uoi.gr/index.php/material-from-publications-software-and-protocols).

In addition to the scenarios described above we also consider an extra scenario representing the ‘typical’ loop; that is a loop with the characteristics most commonly encountered in our collection of 303 loops. We specified this such that one comparison was informed by a single trial and the median number of studies per loop was 8, in line with the empirical evidence. The median loop sample size is 2300 (i.e. average trial arm size 144) [14]. Consequently, a loop with K_AB_ = 1, K_AC_ = 4, K_BC_ = 3, and n ~ U(120, 160) can be considered to be an ‘average sized loop’.

## Results

### Type I error

**Figure 2:**
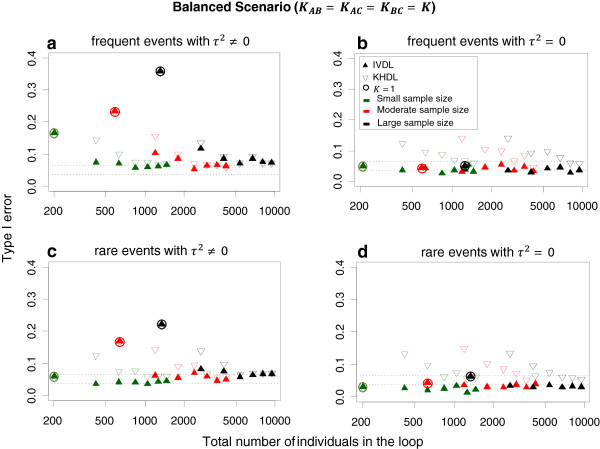
**Type I error by sample sizes, frequency of events and loop sample size.** We assume *equal* number of trials per comparison (K_AB_ = K_AC_ = K_BC_ = K = 1, …, 7) in the presence (τ^2^ ≠ 0) and absence (τ^2^ = 0) of heterogeneity. Circled points correspond to loops with K = 1 for which a fixed-effects model is employed. The region within the horizontal dotted lines defines the confidence interval for the 5% nominal level. IVDL: inverse variance method using the DerSimonian and Laird estimator, KHDL: Knapp-Hartung method with the DerSimonian and Laird estimator.

Figure 2 and Additional file 1: Figure S1 display the estimated type I error for equal and different numbers of trials across comparisons. In general, type I error is close to the nominal level for IVDL, but larger than 5% for many scenarios analysed with KHDL. The KHDL method generally yields smaller variances for IF, leading to larger type I errors (average type I error across all scenarios for IVDL: 0.07, average type I error across all scenarios for KHDL: 0.10, see also Figure 2a and b). Type I error converges to the nominal level more rapidly when τ^2^ = 0 for both IVDL and KHDL methods. The overall type I error approaches the nominal level as the number of trials increases for the same trial size. For example, for frequent events type I error reaches on average the nominal level when K = 5 for small sample sizes, and K = 4 for moderate and large sample sizes. In Table 2 we provide the type I error values for various simulation scenarios. When the total number of individuals included in the network ranges from 2400 to 3000 (i.e. close to the empirically estimated median loop size) type I error lies between 0.06 and 0.08. Type I error deviates from 5% considerably when an equal and small number of trials is considered across comparisons for all trial sizes (see Figure 2a ,b and Table 2).

**Table 2:** **Type I error, power and coverage probability by sample size and number of trials**

	Balanced scenario(K _AB_= K _AC_= K _BC_= K)							Imbalanced scenario
	K = 1	K = 2	K = 3	K = 4	K = 5	K = 6	K = 7	K _AB_=1
								K _AC_=4
								K _BC_=7
Type I error (IF = 0)								
n ~ U(20,50)	0.07	0.07	0.06	0.04	0.05	0.05	0.04	0.06
n ~ U(50,150)	0.10	0.07	0.06	0.06	**0.05**	**0.06**	0.04	**0.08**
n ~ U(150,300)	0.13	**0.07**	0.05	0.06	0.06	0.04	0.05	0.06
Power (IF = 0.6)								
n ~ U(20,50)	0.13	0.15	0.18	0.23	0.27	0.33	0.37	0.16
n ~ U(50,150)	0.25	0.30	0.42	0.52	**0.62**	**0.70**	0.76	**0.32**
n ~ U(150,300)	0.42	**0.54**	0.70	0.79	0.84	0.88	0.89	0.49
Coverage Probability (IF = 0.6)								
n ~ U(20,50)	0.96	0.96	0.97	0.98	0.97	0.97	0.97	0.97
n ~ U(50,150)	0.95	0.96	0.97	0.96	**0.96**	**0.96**	0.96	**0.95**
n ~ U(150,300)	0.93	**0.95**	0.94	0.94	0.96	0.95	0.95	0.95

Results are presented for frequent events and aggregated over different assumptions for heterogeneity and methods to estimate the variances of the mean treatment effects. In bold we present results from loops in which the total number of individuals is between 2400 and 3000. n: sample size, K: number of trials.

For rare events, type I error departs from 5% more than it does for frequent events (Figure 2). Type I error is lower than its nominal level in most cases for IVDL especially when τ^2^ = 0, probably due to overestimation of τ^2^. The KHDL method results again in considerably larger type I errors, which is probably due to the small variances of the mean treatment effects (average type I error across all scenarios for IVDL: 0.05, average type I error across all scenarios for KHDL: 0.08, see Figure 2c and d). Type I error is closer to the nominal level for IVDL when τ^2^ ≠ 0 for all sample sizes. All methods tend to improve their performance with increasing total number of trials included in the entire network (Figure 2 and Additional file 1: Figure S1).

### Statistical power

**Figure 3:**
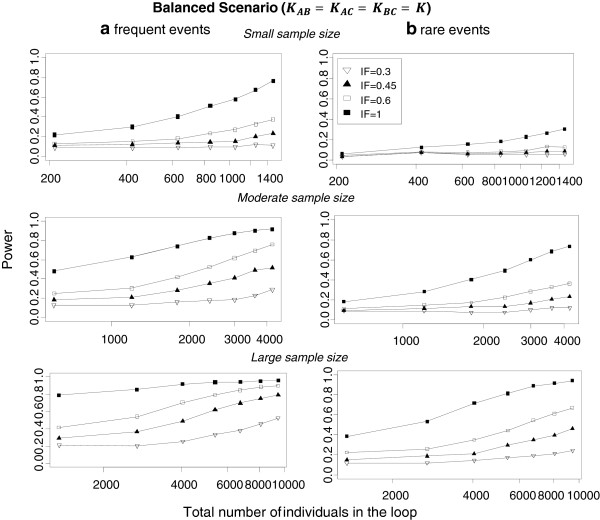
**Power by inconsistency factor, frequency of events and loop sample size.** Power is presented for different sample sizes (small, moderate and large) assuming *equal* number of trials per comparison (K_AB_ = K_AC_ = K_BC_ = K = 1, …, 7). Results are aggregated over different assumptions for heterogeneity and methods to estimate the variance of the mean treatment effect. The first summary result in each power curve pertains to the case where there is a single trial per comparison and a fixed-effects model is employed. IF: inconsistency factor.

Figure 3 and Additional file 2: Figure S2 present the power for IF = {0.3, 0.45, 0.6, 1} for both frequent and rare events when equal (Figure 3) and different (Additional file 2: Figure S2) numbers of trials are included in comparisons. As expected, the overall power increases both with number of trials included in the loop and with the trial size. Power increases when the trials included in a loop have comparable sample sizes. Results are aggregated over all estimation methods for heterogeneity and the different methods to estimate the variance of the direct summary effects. In Table 2 we provide the power values for various simulation scenarios when IF = 0.6 and frequent events are considered. When the total number of individuals included in the network ranges from 2400 to 3000, power ranges between 0.54 and 0.70 when an equal number of trials is assumed across comparisons but drops to 0.32 when each comparison has a different number of trials. As can be seen in equation (2), the distribution of trials across comparisons affects the estimation of inconsistency variance. This has an impact on power and the test is more powerful when trials are distributed uniformly across comparisons. Comparing, for example, the power of the test for the balanced scenario K_AB_ = 4, K_AC_ = 4, K_BC_ = 4 and the imbalanced scenario K_AB_ = 1, K_AC_ = 4, K_BC_ = 7 (each with 12 trials in the loop), power is higher when the distribution of trials is balanced across comparisons (ranges from 0.23 to 0.79) rather than imbalanced (ranges from 0.16 to 0.49) (see Table 2). The comparison of frequent (Figure 3a) and rare (Figure 3b) events indicates that power is larger for frequent events (average power across all scenarios for frequent events: 0.44, average power across all scenarios for rare events: 0.25). Rare events are associated with larger uncertainty for the direct mean treatment effects and thus the chances of identifying potentially important inconsistency decrease. It should be noted that the first summary result of each power curve pertains to the case where there is only one trial per comparison and heterogeneity is set to be zero. This has an impact on monotonicity especially when IF is low and trial size is large.

In Tables 3 and 4 we present the power for IVDL and KHDL methods. For frequent events the power to detect inconsistency does not vary significantly with the method used to estimate heterogeneity or to express uncertainty on the summary effects although the Knapp-Hartung method is marginally more powerful, especially in the absence of heterogeneity. This is because, in many cases, the Knapp-Hartung method estimates smaller inconsistency variances compared with the inverse variance method. The median inconsistency standard error is 0.33 (IQR 0.21, 0.50) for KHDL and 0.40 (IQR 0.27, 0.57) for IVDL. As expected, when there is no heterogeneity, there is less uncertainty associated with each pairwise effect and the power to detect inconsistency increases for all IF values (Table 3).

**Table 3:** **Power of the test for inconsistency aggregated over sample size and number of trials**

	Heterogeneity				No heterogeneity			
	IF = 0.3	IF = 0.45	IF = 0.6	IF = 1	IF = 0.3	IF = 0.45	IF = 0.6	IF = 1
	Frequent Events							
IVDL	0.17	0.26	0.36	0.59	0.20	0.38	0.52	0.77
KHDL	0.19	0.27	0.37	0.60	0.27	0.44	0.58	0.80
	Rare Events							
IVDL	0.10	0.15	0.21	0.38	0.09	0.16	0.25	0.49
KHDL	0.13	0.18	0.24	0.41	0.16	0.23	0.33	0.55

Results are presented for *equal* number of trials across comparisons. IF: inconsistency factor, IVDL: inverse variance method with the DerSimonian and Laird estimator, KHDL: the Knapp-Hartung method with the DerSimonian and Laird estimator.

**Table 4:** **Power of the inconsistency test aggregated over sample size**

	Heterogeneity				No heterogeneity			
	IF = 0.3	IF = 0.45	IF = 0.6	IF = 1	IF = 0.3	IF = 0.45	IF = 0.6	IF = 1
	Frequent Events							
IVDL	0.10	0.15	0.23	0.42	0.13	0.23	0.38	0.68
KHDL	0.11	0.17	0.24	0.42	0.19	0.31	0.44	0.73
	Rare Events							
IVDL	0.08	0.10	0.14	0.25	0.07	0.11	0.17	0.35
KHDL	0.11	0.12	0.16	0.28	0.12	0.17	0.25	0.44

IVDL: inverse variance method with the DerSimonian and Laird estimator, KHDL: Knapp-Hartung method with the DerSimonian and Laird estimator.

The impact of heterogeneity is similar when the outcome is rare (average power across all IF values for KHDL: 0.24, average power across all IF values for IVDL: 0.21, see Table 3). Table 3 shows that the advantage of KHDL method when heterogeneity is zero becomes more pronounced for rare events (average power across all IF values for KHDL: 0.32, average power across all IF values for IVDL: 0.25, see Table 3).

### Coverage probability and bias

We assess how often the 95% CI for inconsistency includes the assumed IF value used to generate the data. We plot the coverage probability for the 95% CI of IF in Additional file 3: Figure S3. The coverage probability is close to the nominal level (95%) for most settings. Rare events are associated with larger uncertainty and therefore provide slightly higher coverage than frequent events (average coverage across all scenarios for frequent events: 0.95, average coverage across all scenarios for rare events: 0.97). In Table 2 we provide the coverage values for various simulation scenarios when IF = 0.6. When the total number of individuals included in the network ranges from 2400 to 3000, coverage ranges from 0.95 to 0.96 (Table 2). Coverage does not change considerably when an equal or different number of trials is assumed across comparisons (Additional file 4: Figure S4).

In Additional file 5: Figure S5 and Additional file 6: Figure S6 we present the average relative bias  for IF > 0. Relative bias decreases with the total number of individuals included in the network, the total number of trials, and the assumed IF value.

Tables 5 and 6 present the coverage probability for the 95% CI of IF using different methods to express uncertainty on the summary effects. The KHDL method reduces slightly the chances of including the true inconsistency factor in the 95% CI of IF, especially when there is no heterogeneity, as the mean treatment effects become more precise.

**Table 5:** **Coverage probability of the 95% confidence interval for the inconsistency factor (IF)**

	Heterogeneity					No heterogeneity				
	IF = 0	IF = 0.3	IF = 0.45	IF = 0.6	IF = 1	IF = 0	IF = 0.3	IF = 0.45	IF = 0.6	IF = 1
Frequent Events										
IVDL	0.90	0.94	0.94	0.94	0.93	0.96	0.98	0.97	0.97	0.97
KHDL	0.89	0.93	0.93	0.93	0.91	0.92	0.95	0.94	0.94	0.93
Rare Events										
IVDL	0.93	0.96	0.96	0.97	0.96	0.97	0.98	0.99	0.98	0.96
KHDL	0.91	0.95	0.95	0.95	0.94	0.92	0.96	0.96	0.95	0.94

Results are aggregated over sample size and number of trials (assumed *equal* across comparisons). IVDL: inverse variance method with the DerSimonian and Laird estimator, KHDL: Knapp-Hartung method with the DerSimonian and Laird estimator.

**Table 6:** **Coverage probabilities of the 95% confidence interval for the inconsistency factor (IF)**

	Heterogeneity					No heterogeneity				
	IF = 0	IF = 0.3	IF = 0.45	IF = 0.6	IF = 1	IF = 0	IF = 0.3	IF = 0.45	IF = 0.6	IF = 1
Frequent Events										
IVDL	0.92	0.96	0.96	0.96	0.95	0.97	0.98	0.98	0.97	0.97
KHDL	0.91	0.95	0.96	0.95	0.94	0.93	0.96	0.95	0.95	0.93
Rare Events										
IVDL	0.95	0.96	0.97	0.98	0.98	0.97	0.98	0.98	0.99	0.99
KHDL	0.93	0.95	0.96	0.96	0.96	0.93	0.96	0.96	0.96	0.95

IVDL: inverse variance method with the DerSimonian and Laird estimator, KHDL: Knapp-Hartung method with the DerSimonian and Laird estimator.

### Characteristics of the inconsistency test in a ‘typical’ loop of evidence

The type I error in the ‘typical’ loop is 5% and 7% for subjective and all-cause mortality outcomes using IVDL and 11% and 12% using KHDL. The ‘typical’ loop of evidence with all-cause mortality outcome has considerably low power. The overall power ranges between 14% and 75% for IVDL and 21% to 78% for KHDL depending on the magnitude of inconsistency. For a subjective outcome that pertains to larger heterogeneity power decreases to 14%-63% for IVDL and in 20% to 65% for KHDL. Coverage is close to the 95% nominal level (see Table 7).

**Table 7:** **Type I error, power and coverage probability for the inconsistency test in a ‘typical’ loop of evidence**

	Type I error	Power				Coverage probability				
	IF = 0	IF = 0.3	IF = 0.45	IF = 0.6	IF = 1	IF = 0	IF = 0.3	IF = 0.45	IF = 0.6	IF = 1
	All-cause mortality outcome (median (τ^2^) = 0.02)									
IVDL	0.05	0.14	0.23	0.38	0.75	0.95	0.97	0.99	0.98	0.95
KHDL	0.11	0.21	0.32	0.46	0.78	0.89	0.94	0.93	0.92	0.90
	Subjective outcome (median (τ^2^) = 0.11)									
IVDL	0.07	0.14	0.23	0.34	0.63	0.94	0.96	0.96	0.97	0.95
KHDL	0.12	0.20	0.29	0.41	0.65	0.88	0.93	0.93	0.92	0.91

We assume a dichotomous frequent outcome, number of trials (K) per comparison K_AB_ = 1, K_AC_ = 4, K_BC_ = 3 and the sample size per arm is drown from n **~** U(120, 160). IF: inconsistency factor, IVDL: inverse variance method with the DerSimonian and Laird estimator, KHDL: Knapp-Hartung method with the DerSimonian and Laird estimator.

## Discussion

The increased use of network meta-analysis should be accompanied by caution when combining direct and indirect evidence via careful assessment of the consistency assumption. Protocols of network meta-analyses should present methods for the evaluation of inconsistency and define strategies to be followed when inconsistency is present. Several methodologies have been outlined in the literature to test inconsistency [4–9]. In this study, we evaluate the properties of the z-test for detecting inconsistency comparing direct and indirect estimates in triangular networks generating 1000 loops for each scenario presented in Table 1. Although running more than 1000 simulations per scenario would have decreased the Monte Carlo error, we believe the main conclusions from our simulations are robust. Our scenarios are informed by previous large-scale empirical studies and hence are directly applicable [14, 22]. We use a variety of scenarios that involve the most commonly used meta-analytic tools for statistical inference regarding heterogeneity and the uncertainty of the mean treatment effects. The main advantage of this work is that it sheds light on factors that might affect the detection of inconsistency and have not been examined in the past, such as the use of Knapp-Hartung variance for the direct summary effects. Our main findings are summarized below.

- The assumption of consistency in network meta-analysis is often evaluated performing a z-test within each loop of evidence.
- The inconsistency test has low power for the ‘typical’ loop (comprising 8 trials and about 2000 participants) found in published networks. This study suggests that the probability to detect inconsistency when present is between 14% and 21% depending on the estimation method.
- Power is positively associated with sample size and frequency of the outcome, and negatively associated with the underlying extent of heterogeneity.
- Using the Knapp-Hartung method to estimate uncertainty around meta-analytic effects is slightly more powerful than the inverse variance approach.
- Type I error converges to the nominal level as the total number of individuals included in the loop increases while coverage is close to the nominal level for most studied scenarios.
- We recommend that investigators a) employ a variety of methods to evaluate inconsistency, b) interpret the magnitude of the estimated inconsistency factor and its confidence interval c) adopt a sceptical stance towards statistically non-significant test results unless the loop of evidence has many data d) always consider the comparability of the studies in terms of potential effect modifiers to infer about the possibility of inconsistency

Our simulation study shows that the inconsistency test has on average low power to detect inconsistency, in particular for rare outcomes (i.e. for IF = 0.3 and large trial sizes a rare outcome has event rate on average 0.10 IQR (0.07, 0.13)). Bruadbrn et al. [31] state that the IVDL method may be “unsuitable when there are few events” and that it should be avoided. In the absence of heterogeneity and for a large number and size of trials the overall power for inconsistency might be adequate. A previous simulation study [15] also found that different ways to evaluate inconsistency (e.g. Lu and Ades [6] model, node-splitting method [9]) have low power in particular under the random-effects models. Our study suggests that power is improved if the Knapp-Hartung method is used, especially in the absence of heterogeneity, although the type I error increases as well. This is because the estimated uncertainty around inconsistency is small with Knapp-Hartung method. These findings agree with a previous simulation study, which showed that when heterogeneity is zero the Knapp-Hartung method yields a smaller variance for the mean treatment effects than the inverse variance method [21].

Several methods have been suggested to estimate heterogeneity τ^2^ [32, 33]. In the present study we also included the restricted maximum likelihood [34] and the empirical Bayes [35] estimators in conjunction with the inverse variance approach. Although the three estimators have different properties and performance in general, they have been showed to have comparable bias and mean squared error for estimating τ^2^ in the examined simulation scenarios (relatively small number of trials for each pairwise meta-analysis (fewer than 7) and median heterogeneity τ^2^ = 0.12 are comparable [32]. Consequently type I error, power and coverage were found similar between the three methods (data not shown) and we present results only from IVDL and KHDL. This agrees with an empirical study that compared five different estimators for the heterogeneity and showed that variability in the confidence intervals of the overall treatment effect was quite negligible across 920 Cochrane meta-analyses [36].

The inconsistency test, analogously to the heterogeneity test, has low power and we recommend that the point estimate of inconsistency and its 95% confidence interval are used instead to draw inferences about the presence and magnitude of inconsistency. In cases where the test is underpowered, the confidence intervals would include zero, small and large inconsistency values and should be interpreted as lack of evidence for or against the presence of inconsistency. If a test must be used, one possibility is to use a cut-off p-value of 0.10, as has been suggested for the heterogeneity test in pairwise meta-analysis [37, 38]. Empirical evidence showed that the observed disagreement between direct and indirect comparisons is 1 in 10 loops, so this cut-point might be a reasonable choice [14]. In complex networks, instead of using multiple underpowered z-test, global tests such as the design-by-treatment test can be used, although power properties of the latter are unknown.

Some limitations in our study need to be acknowledged. We do not account for the possible impact of multi-arm trials on inconsistency and we only reconsider triangular networks. Our previous empirical study showed that a large majority (85%) of published networks of interventions involve trials with multiple arms, and that out of the total 1173 trials included in all 40 networks 116 (10%) were multi-arm trials. Further simulation studies are therefore needed to evaluate complex networks with multi-arm trials. In our simulation study we assume that all comparisons in the network share the same amount of heterogeneity. Turner et al. [22] showed that different amounts of heterogeneity can be expected for different outcomes or for different classes of interventions (e.g. pharmacological vs. non-pharmacological). Network meta-analyses typically consider only one outcome and often compare interventions of a similar nature. Hence the assumption of equal heterogeneities is often clinically reasonable as well as being statistically convenient. Most comparisons in networks comprise only few studies, making estimation of heterogeneity challenging. In case heterogeneity is believed to vary across comparisons, we can assume different parameters which should be restricted to conform to special relationships according to the consistency assumption [39]. Finally, a thorough investigation of all available methods to evaluate inconsistency using realistic scenarios informed by empirical evidence would be needed for completeness [5–7].

This is the second simulation study that suggests statistical evaluation of inconsistency has low power [15]. In our simulations we consider three-treatment networks for simplicity but analyse them using methods typically employed for network meta-analysis, e.g. assuming common heterogeneity in a one-stage analysis. As inconsistency is a property of a closed loop, we believe that our results are very relevant to full networks. Although our study is limited to simple three-treatment networks including only two-arm trials, we anticipate that the inconsistency test would show similarly low power in the presence of multi-arm studies: such studies are internally consistent and would contribute similar pairwise comparisons to evaluations of inconsistency. Further simulation studies might be needed to learn about the impact of assuming different heterogeneity parameters for different comparisons. Reliable estimation of different heterogeneity parameters will require a minimum number of studies for each comparison, a scenario which seldom occurs in published networks of interventions. The Knapp-Hartung method has been shown to be robust to the estimation of heterogeneity [21] so we suspect that conclusions would be similar to those drawn from the present study. It is therefore imperative for investigators to evaluate the assumption of consistency using epidemiological strategies and compare carefully the involved studies with respect to the distribution of effect modifiers before embarking into data synthesis [3, 40].

## Conclusions

Although the performance of the z-test for inconsistency might vary according to the method used to estimate the uncertainty of the overall mean treatment effect, the power remains generally low for the loop of evidence that typically features in networks of interventions. Particularly when data is sparse and a loop includes only a few studies or the outcome is rare, the inconsistency test is unlikely to be informative.

## Electronic supplementary material

Additional file 1: Figure S1: Type I error by sample sizes, frequency of events and loop sample size. Results are shown assuming *different* number of trials (K) per comparison (K_AB_ = 1, K_AC_ = 4, K_BC_ = 7). The region within the horizontal dotted lines defines the confidence interval for the 5% nominal level. IVDL: inverse variance method using the DerSimonian and Laird estimator, KHDL: Knapp-Hartung method with the DerSimonian and Laird estimator. (PPTX 113 KB)

Additional file 2: Figure S2: Power by inconsistency factor, frequency of events and loop sample size. We assume *different* number of trials (K) per comparison (K_AB_ = 1, K_AC_ = 4, K_BC_ = 7). Results are aggregated over different assumptions for the heterogeneity and methods to estimate the variances of the mean treatment effects. IF: inconsistency factor. (PPTX 80 KB)

Additional file 3: Figure S3: Coverage probabilities of the 95% confidence interval for the inconsistency factor, frequency of events and loop sample size. We assume *equal* number of trials per comparison (K_AB_ = K_AC_ = K_BC_ = K = 1, …, 7). Results are aggregated over different assumptions for the heterogeneity and methods to estimate the variances of the mean treatment effects. The region within the horizontal dotted lines defines the confidence interval for the 95% nominal level. The first summary result in each coverage probability line pertains to the case where there is a single trial per comparison and a fixed-effects model is employed. (PPTX 145 KB)

Additional file 4: Figure S4: Coverage probabilities of the 95% confidence interval for the inconsistency factor (IF), frequency of events and loop sample size. We assume *different* number of trials (K) per comparison (K_AB_ = 1, K_AC_ = 4, K_BC_ = 7). Results are aggregated over different assumptions for the heterogeneity and methods to estimate the variances of the mean treatment effects. The region within the horizontal dotted lines defines the confidence interval for the 95% nominal level. (PPTX 87 KB)

Additional file 5: Figure S5: Averaged relative bias assuming various scenarios for the inconsistency factor, the frequency of events and loop sample size. We assume *equal* number of trials per comparison (K_AB_ = K_AC_ = K_BC_ = K = 1, …, 7). Results are aggregated over different assumptions for the heterogeneity and methods to estimate the variances for the direct treatment effects. IF: inconsistency factor. (PPTX 127 KB)

Additional file 6: Figure S6: Averaged relative bias assuming various scenarios for the inconsistency factor, the frequency of events and loop sample size. We assume *different* number of trials (K) per comparison (K_AB_ = 1, K_AC_ = 4, K_BC_ = 7). Results are aggregated over different assumptions for the heterogeneity and methods to estimate the variances of the mean treatment effects. IF: inconsistency factor. (PPTX 83 KB)

## Abbreviations

CI: Confidence interval
DIR: Direct
IF: Inconsistency factor
IND: Indirect
IVDL: Inverse variance method using DerSimonian and Laird estimator
IQR: Interquantile range; method
KHDL: Knapp-Hartung method using DerSimonian and Laird estimator
LOR: Log-odds ratio
OR: Odds ratio.
