# Supplementary material for: Characteristics of a loop of evidence that affect detection and estimation of inconsistency: a simulation study
Source: BMC Med Res Methodol. 2014 Sep 19;14:106. doi: 10.1186/1471-2288-14-106 (PMC4190337; doi:10.1186/1471-2288-14-106)
Supplement: Supplementary file 1 — Additional file 1: Figure S1: Type I error by sample sizes, frequency of events and loop sample size. Results are shown assuming different number of trials (K) per comparison (KAB = 1, KAC = 4, KBC = 7). The region within the horizontal dotted lines defines the confidence interval for the 5% nominal level. IVDL: inverse variance method using the DerSimonian and Laird estimator, KHDL: Knapp-Hartung method with the DerSimonian and Laird estimator. (PPTX 113 KB) [file 12874_2013_1120_MOESM1_ESM.pptx]

## Slide 1
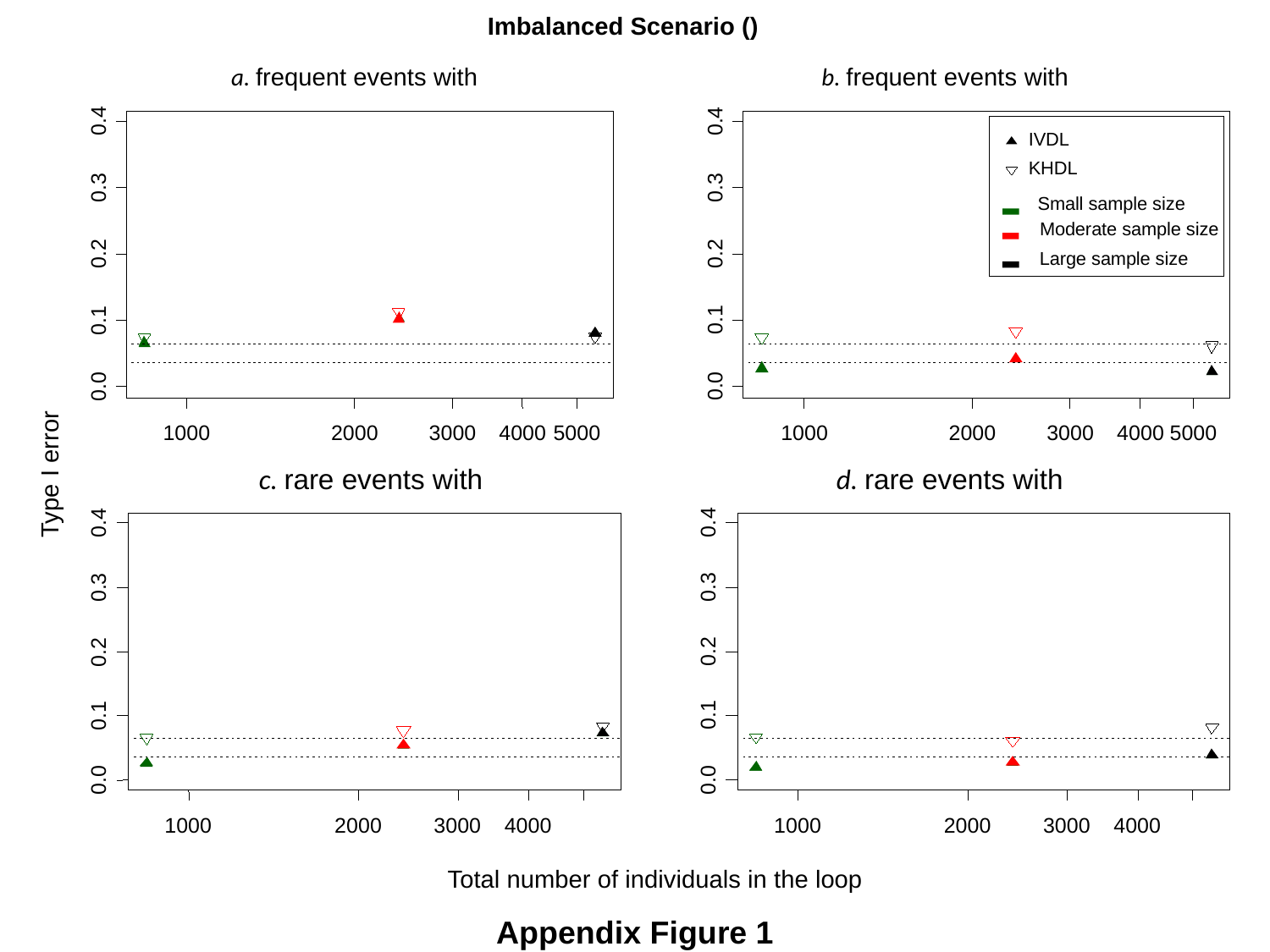

0.4
0.3
0.2
0.1
0.0
1000
2000
3000
4000
5000
0.4
0.3
0.2
0.1
0.0
1000
2000
3000
4000
5000
Type I error
0.4
0.3
0.2
0.1
0.0
1000
2000
3000
4000
0.4
0.3
0.2
0.1
0.0
1000
2000
3000
4000
Total number of individuals in the loop
IVDL
KHDL
Small sample size
Moderate sample size
Large sample size
Appendix Figure 1
