# Supplementary material for: Characteristics of a loop of evidence that affect detection and estimation of inconsistency: a simulation study
Source: BMC Med Res Methodol. 2014 Sep 19;14:106. doi: 10.1186/1471-2288-14-106 (PMC4190337; doi:10.1186/1471-2288-14-106)
Supplement: Supplementary file 2 — Additional file 2: Figure S2: Power by inconsistency factor, frequency of events and loop sample size. We assume different number of trials (K) per comparison (KAB = 1, KAC = 4, KBC = 7). Results are aggregated over different assumptions for the heterogeneity and methods to estimate the variances of the mean treatment effects. IF: inconsistency factor. (PPTX 80 KB) [file 12874_2013_1120_MOESM2_ESM.pptx]

## Slide 1
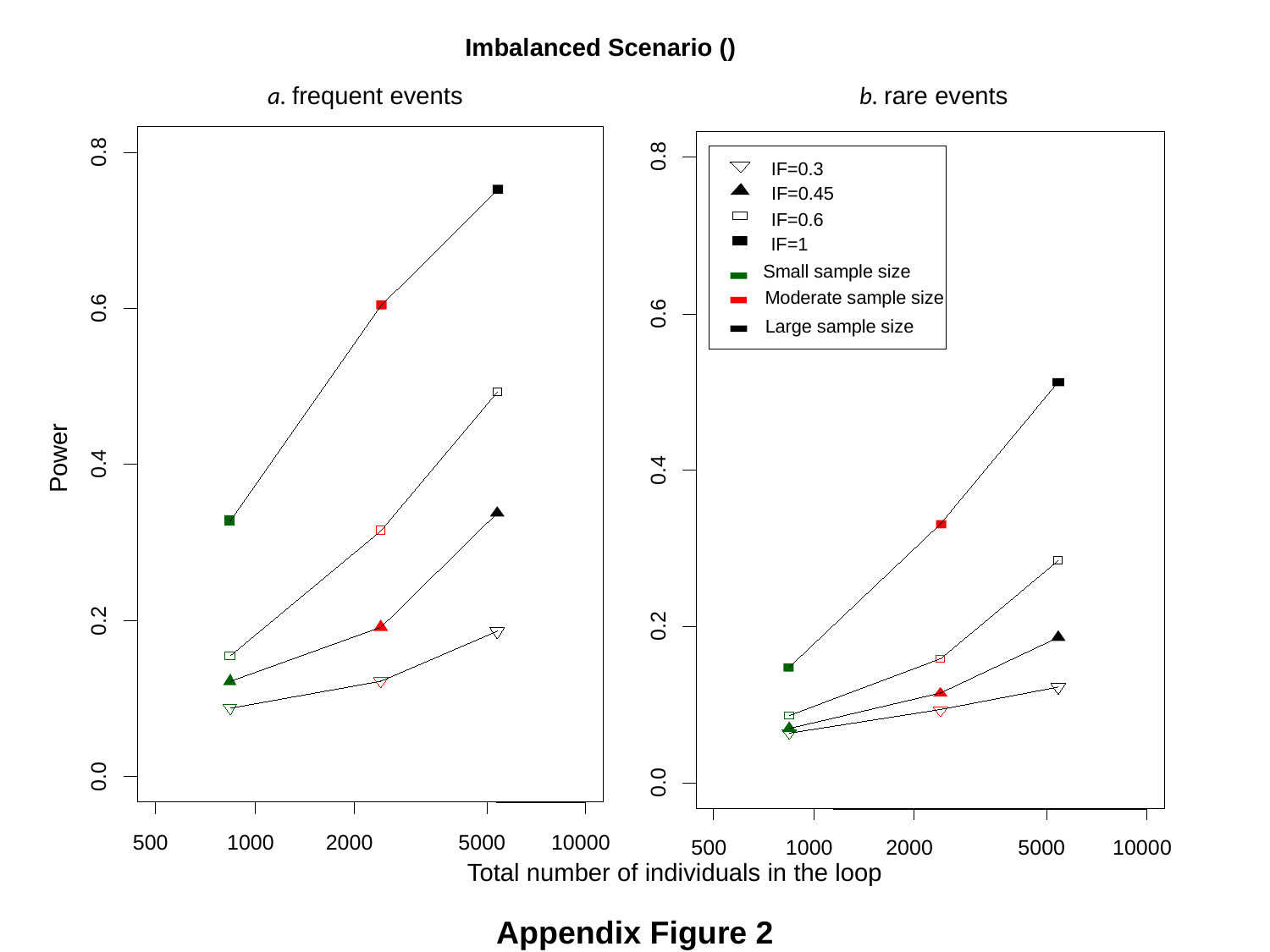

a. frequent events
b. rare events
0.8
0.6
0.4
0.2
0.0
500
1000
2000
5000
10000
0.8
0.6
0.4
0.2
0.0
500
1000
2000
5000
10000
IF=0.3
IF=0.45
IF=0.6
IF=1
Small sample size
Moderate sample size
Large sample size
Power
Total number of individuals in the loop
Appendix Figure 2
