# Supplementary material for: Characteristics of a loop of evidence that affect detection and estimation of inconsistency: a simulation study
Source: BMC Med Res Methodol. 2014 Sep 19;14:106. doi: 10.1186/1471-2288-14-106 (PMC4190337; doi:10.1186/1471-2288-14-106)
Supplement: Supplementary file 3 — Additional file 3: Figure S3: Coverage probabilities of the 95% confidence interval for the inconsistency factor, frequency of events and loop sample size. We assume equal number of trials per comparison (KAB = KAC = KBC = K = 1, …, 7). Results are aggregated over different assumptions for the heterogeneity and methods to estimate the variances of the mean treatment effects. The region within the horizontal dotted lines defines the confidence interval for the 95% nominal level. The first summary result in each coverage probability line pertains to the case where there is a single trial per comparison and a fixed-effects model is employed. (PPTX 145 KB) [file 12874_2013_1120_MOESM3_ESM.pptx]

## Slide 1
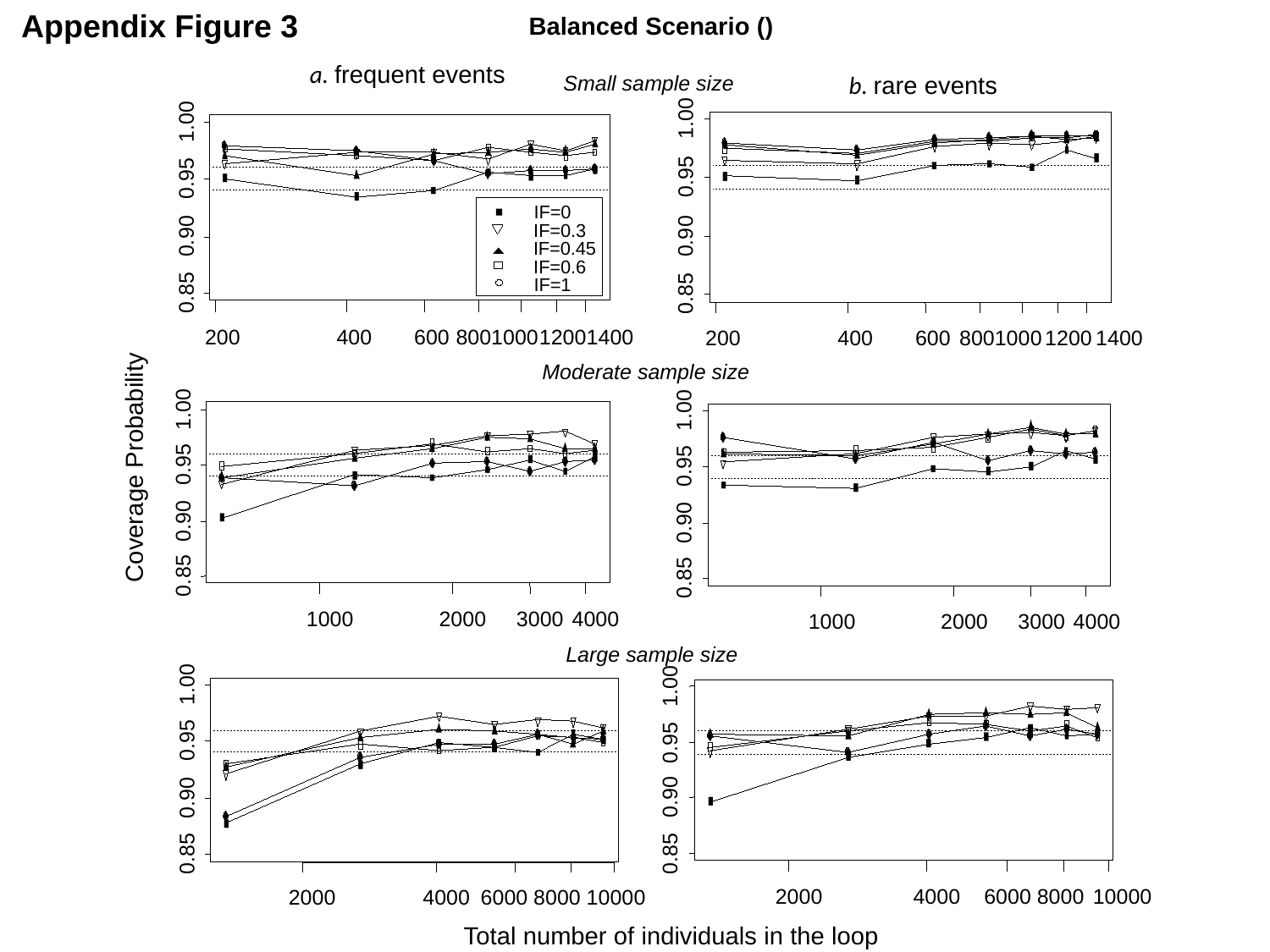

Appendix Figure 3
a. frequent events
b. rare events
Small sample size
1.00
0.95
0.90
0.85
200
400
600
800
1000
1200
1400
1.00
200
400
600
800
1000
1200
1400
0.95
0.90
0.85
Moderate sample size
1.00
0.95
0.90
0.85
1000
2000
3000
4000
1.00
0.95
0.90
0.85
1000
2000
3000
4000
Coverage Probability
Large sample size
1.00
0.95
0.90
0.85
2000
4000
6000
8000
10000
1.00
0.95
0.90
0.85
2000
4000
6000
8000
10000
Total number of individuals in the loop
IF=0
IF=0.3
IF=0.45
IF=0.6
IF=1
