# Supplementary material for: Characteristics of a loop of evidence that affect detection and estimation of inconsistency: a simulation study
Source: BMC Med Res Methodol. 2014 Sep 19;14:106. doi: 10.1186/1471-2288-14-106 (PMC4190337; doi:10.1186/1471-2288-14-106)
Supplement: Supplementary file 4 — Additional file 4: Figure S4: Coverage probabilities of the 95% confidence interval for the inconsistency factor (IF), frequency of events and loop sample size. We assume different number of trials (K) per comparison (KAB = 1, KAC = 4, KBC = 7). Results are aggregated over different assumptions for the heterogeneity and methods to estimate the variances of the mean treatment effects. The region within the horizontal dotted lines defines the confidence interval for the 95% nominal level. (PPTX 87 KB) [file 12874_2013_1120_MOESM4_ESM.pptx]

## Slide 1
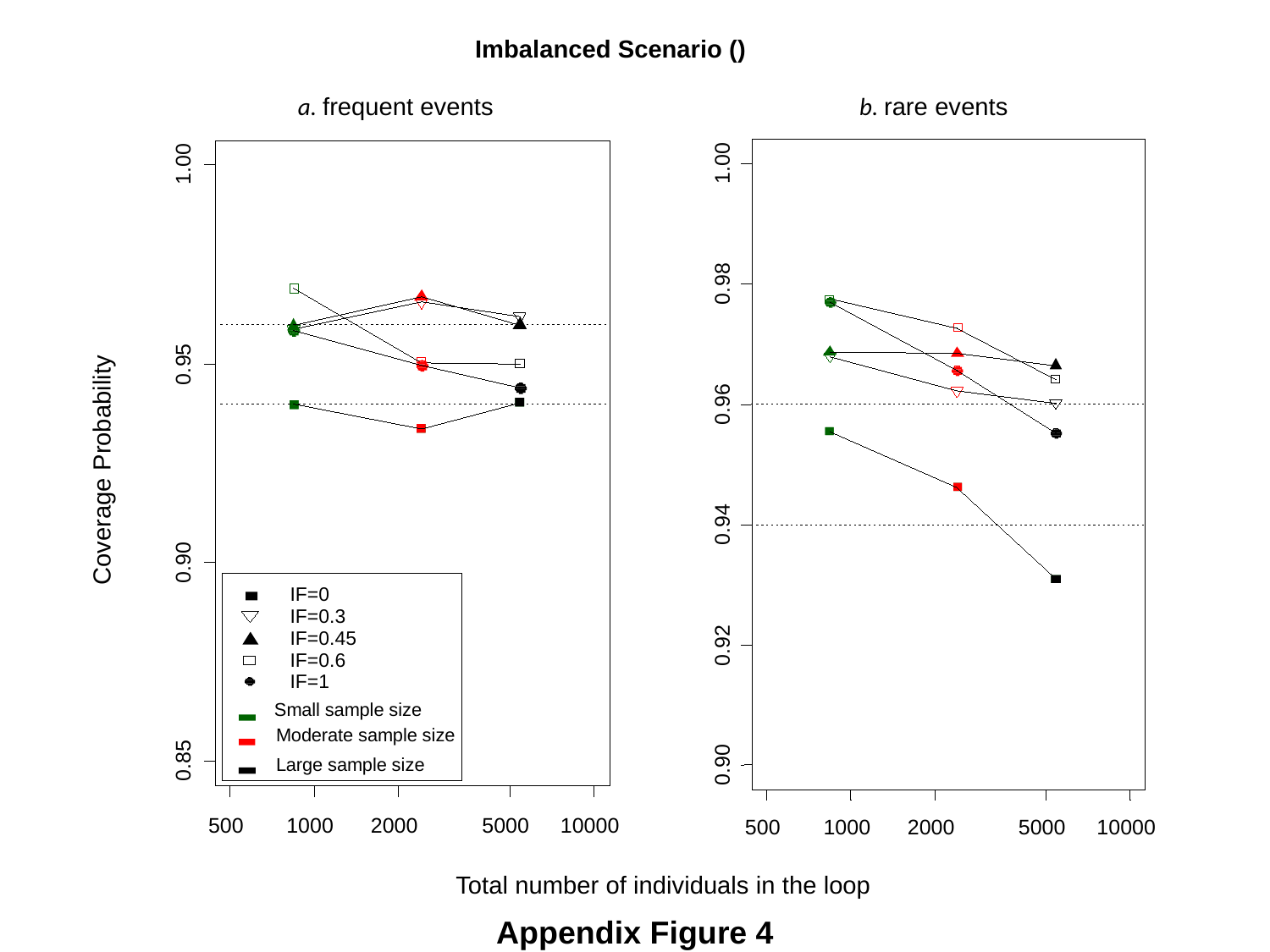

a. frequent events
b. rare events
1.00
0.98
0.96
0.94
0.92
0.90
500
1000
2000
5000
10000
1.00
0.95
0.90
0.85
500
1000
2000
5000
10000
Coverage Probability
IF=0
IF=0.3
IF=0.45
IF=0.6
IF=1
Small sample size
Moderate sample size
Large sample size
Total number of individuals in the loop
Appendix Figure 4
