# Supplementary material for: Characteristics of a loop of evidence that affect detection and estimation of inconsistency: a simulation study
Source: BMC Med Res Methodol. 2014 Sep 19;14:106. doi: 10.1186/1471-2288-14-106 (PMC4190337; doi:10.1186/1471-2288-14-106)
Supplement: Supplementary file 5 — Additional file 5: Figure S5: Averaged relative bias assuming various scenarios for the inconsistency factor, the frequency of events and loop sample size. We assume equal number of trials per comparison (KAB = KAC = KBC = K = 1, …, 7). Results are aggregated over different assumptions for the heterogeneity and methods to estimate the variances for the direct treatment effects. IF: inconsistency factor. (PPTX 127 KB) [file 12874_2013_1120_MOESM5_ESM.pptx]

## Slide 1
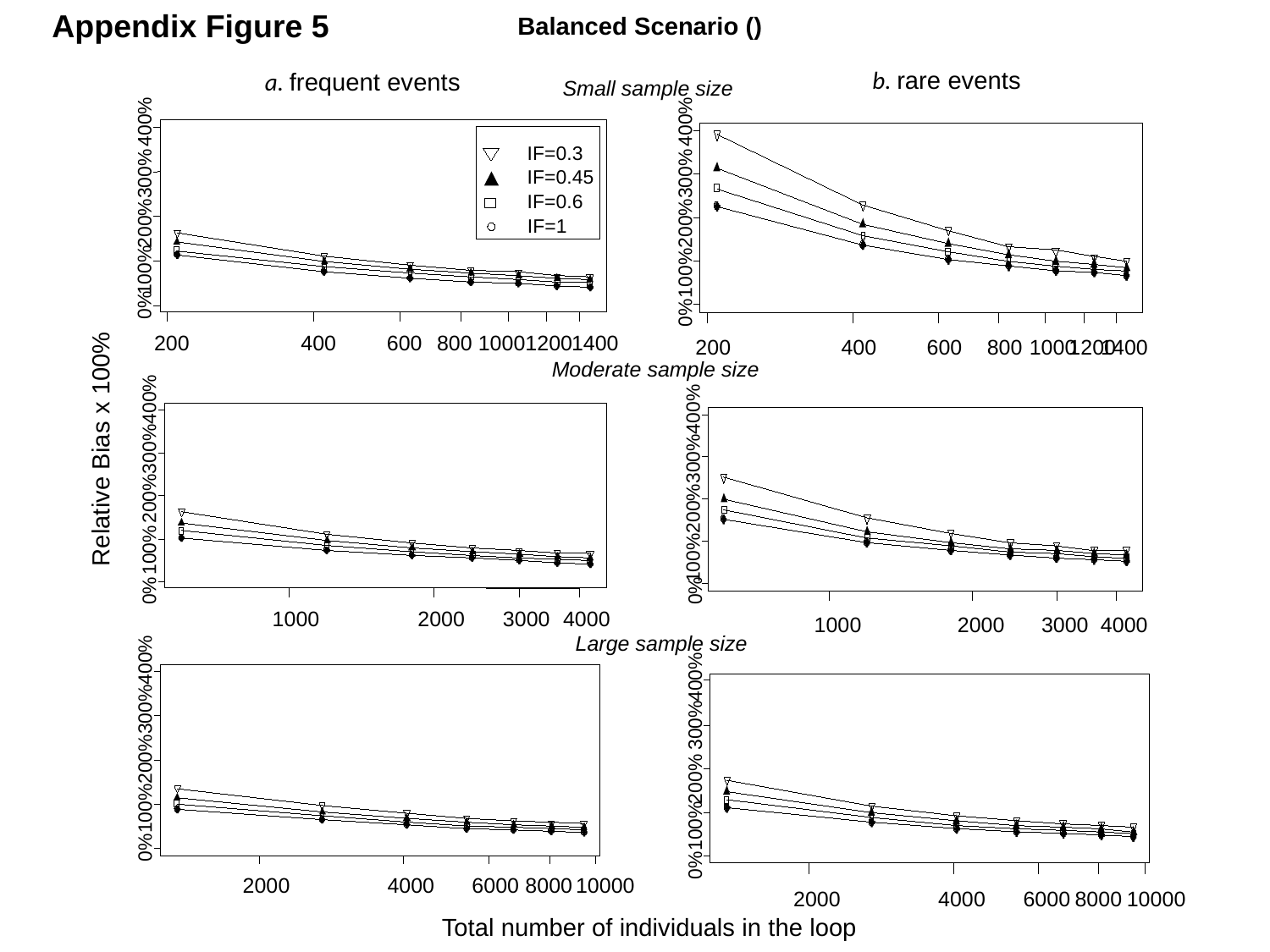

Appendix Figure 5
b. rare events
a. frequent events
Small sample size
400%
300%
200%
100%
0%
200
400
600
800
1000
1200
1400
400%
300%
200%
100%
0%
200
400
600
800
1000
1200
1400
Moderate sample size
400%
300%
200%
100%
0%
1000
2000
3000
4000
400%
300%
200%
100%
0%
1000
2000
3000
4000
Relative Bias x 100%
Large sample size
400%
300%
200%
100%
0%
2000
4000
6000
8000
10000
400%
300%
200%
100%
0%
2000
4000
6000
8000
10000
Total number of individuals in the loop
IF=0.3
IF=0.45
IF=0.6
IF=1
